# ‘Malignant granular cell tumor of the Appendiceal orifice: a rare case and review of literature’

**DOI:** 10.1093/omcr/omaf190

**Published:** 2025-10-22

**Authors:** Omer Usman, Muhammad Waqas Khan, Maham Tariq, Muhammad Usman Shahbaz, Marwa Javed Warriach, Muhammad Abdul Basit, Hassan Shah Aryani

**Affiliations:** Department of Internal Medicine, Texas Tech University Health Sciences Center El Paso, The Hospital of Providence-2000 Transmountain Road, TX 79911, United States; Department of Internal Medicine, MUSC Health Lancaster Medical Center, 800 W Meeting St, SC 29720, United States; Department of Internal Medicine, Services Institute of Medical Sciences, Jail Road, Shadman 54000, Lahore, Pakistan; Department of Internal Medicine, Jinnah Hospital, Usmani Rd, Faisal Town, Lahore 54550, Pakistan; Department of Internal Medicine, Services Institute of Medical Sciences, Jail Road, Shadman 54000, Lahore, Pakistan; Department of Internal Medicine, UHS Wilson Medical Center, Harrison St. Johnson City, NY 13790, United States; Department of Internal Medicine, S. Tenteshiv Memorial Asain Medical Institute, Kant 725012, Kyrgyzstan

**Keywords:** case report, granular cell tumor, appendix, colonoscopy

## Abstract

Granular cell tumors (GCTs) are rare, mostly benign soft-tissue neoplasms, with only 8% occurring in the gastrointestinal tract, making the colon an uncommon location. We present the case of a 37-year-old Hispanic woman who underwent a screening colonoscopy due to a family history of colon cancer. A submucosal nodule at the appendiceal orifice was resected, and a biopsy revealed a malignant GCT, confirmed by S100-positive staining. The patient underwent a right hemicolectomy, with the resection showing a 2 mm focus of granular cell tumor with clear margins. Histopathology confirmed malignancy, with additional findings of reactive hyperplasia of mucosa-associated lymphoid tissue and focal ileitis. Although rare in the colon, malignant GCTs carry a high risk of mortality and recurrence. Small lesions (< 1 cm) can be monitored, but larger or malignant ones require surgical resection. Our case emphasizes the importance of early detection and intervention to prevent severe outcomes in malignant GCTs.

## Introduction

Granular cell tumors (GCTs), also known as Abrikossoff tumors, are relatively uncommon neoplasms originating from Schwann cells. Approximately 150 cases of colonic granular cell tumors have been reported in the English-language literature thus far [1]. The highest incidence is observed between 40 and 60 years of age, with a slight female predominance and higher prevalence in Black individuals. The head and neck region is involved in about 45–65% of patients, while the gastrointestinal (GI) tract is affected in approximately 8% of case [2]. Of GI involvement, 5–10% originate in the digestive tract, and around 1% arise from the appendix [1]. We describe the first reported case of a malignant GCT at the appendiceal orifice in an asymptomatic 37-year-old Hispanic woman undergoing routine screening colonoscopy. The aim of this study is to highlight the importance of surgical resection as an effective therapy, especially in the early stage of the disease.

### Case report

**Figure 1:**
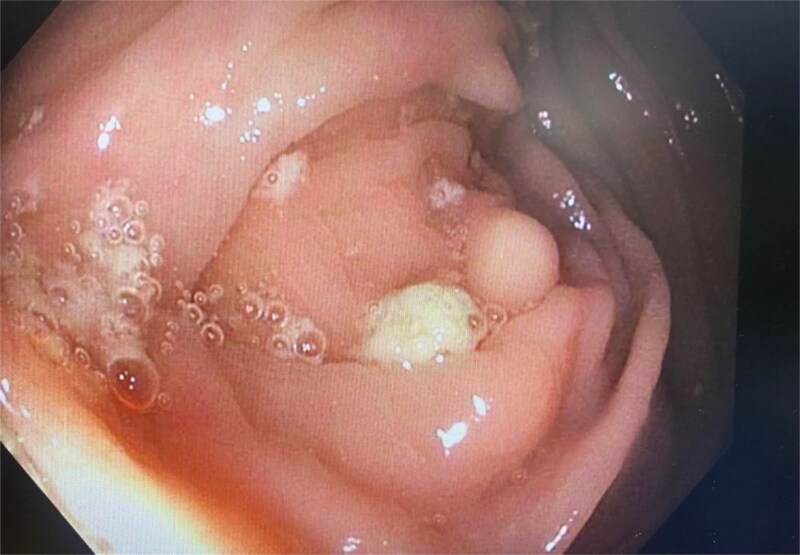
Colonoscopy showing nodule at appendiceal orifice.

**Figure 2:**
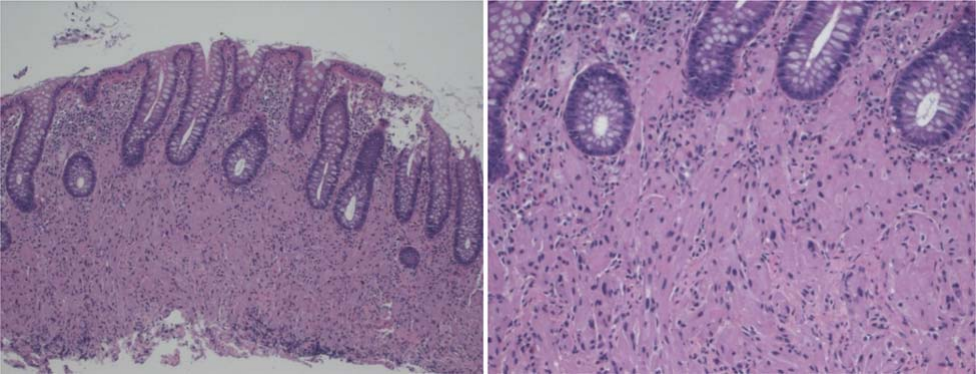
Biopsy shows proliferation of bland round to ovoid cells with prominent eosinophilic, granular cytoplasm.

**Figure 3:**
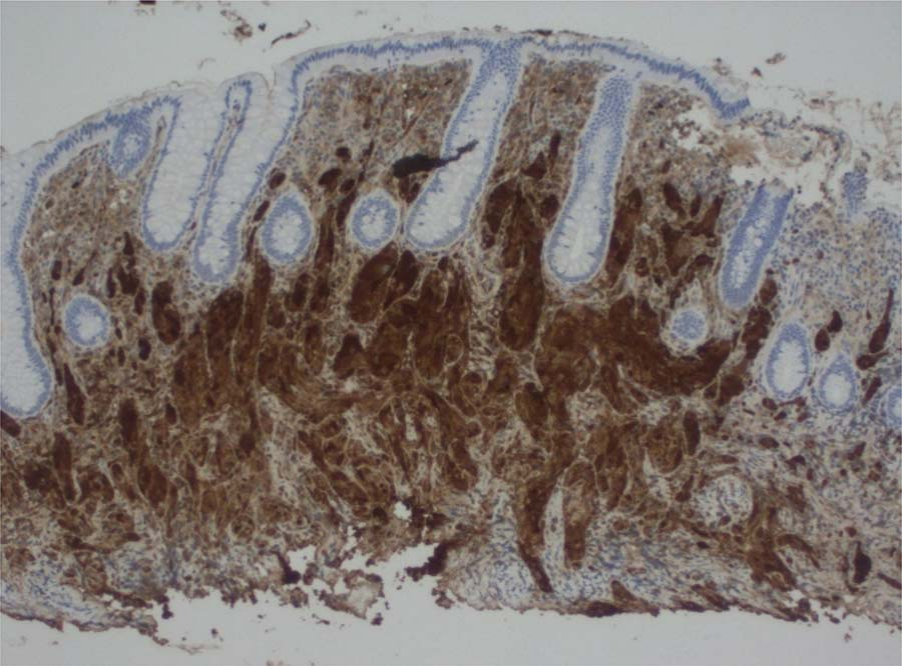
Immunostain shows cells are diffusely positive for S100.

**Figure 4:**
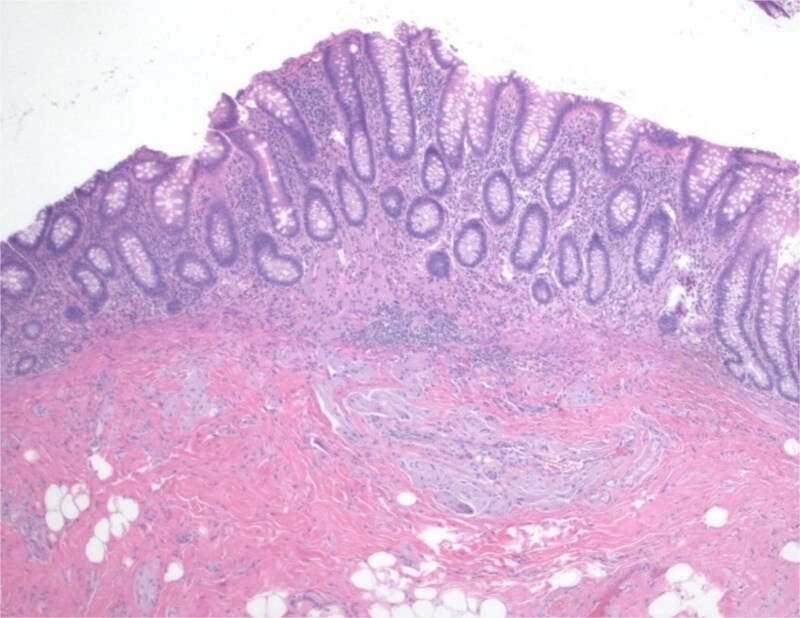
Resection specimen shows focus of granular cell tumor involving mucosa and submucosa.

This 37-year-old Hispanic woman presented for a routine screening colonoscopy due to a strong family history of colon cancer in her mother at age 38. The patient had no past medical history and was not taking any regular medications. Her baseline biochemistry results were within normal limits (Table 2). She was asymptomatic, with no gastrointestinal complaints, and her comprehensive GI examination prior to the procedure was unremarkable. She denied smoking and alcohol intake. During the colonoscopy, a single erosion in the terminal ileum and a submucosal nodule at the appendiceal orifice were noted (Fig. 1). Consequently, the patient was admitted to our hospital for further diagnosis and treatment. A chest computed tomography (CT) scan revealed a faint ‘tree-in-bud’ tiny nodule in the right upper lobe, likely a sequela of infection or an inflammatory process. An abdominal CT scan was normal. The submucosal nodule was resected, and histopathological examination showed a proliferation of round-to-ovoid eosinophilic cells with granular cytoplasm located in the mucosa, submucosa, and lamina propria, consistent with malignancy (Figs 2, 4). Immunohistochemical staining demonstrated the following results: S-100 protein (+), smooth muscle actin (−), desmin (−), CD117 (−), CD34 (−), CD68 (−), neuron-specific enolase (−), and SOX10 (−) (Fig. 3). Given this unusual finding and concern for potential malignancy, the patient was advised to undergo early, aggressive intervention with a right hemicolectomy to prevent fatal outcomes. The resection specimen revealed a 2 mm focus of granular cell tumor with clear resection margins, indicating complete excision. Additionally, reactive hyperplasia of mucosa-associated lymphoid tissue (MALT) and focal ileitis with ulceration were evident upon further tissue analysis. The detection of reactive MALT hyperplasia and focal ileitis with ulceration suggested additional, albeit benign, pathology that required no further intervention beyond monitoring. Postoperatively, the patient was closely monitored with regular clinical assessments, periodic contrast-enhanced CT imaging of the abdomen and pelvis, and follow-up colonoscopy to ensure no recurrence or new pathology.

**Table 1:** 

Sr. no.	Author/year	Gender/Age	Presentation	Tumor size	Tumor location	Tumor nodule	Ref. no.
1	Dhruv et al. 2023	M/55	Hemorrhoids	1 cm	Ascending colon	single	3
2	D Allison et al. 2022	F/58	Acute appendicitis	Not reported	Not reported	single	4
3	Madeira JI et al. 2022	F/30	Pelvic inflammation with pregnancy	0.5 cm	Tip of appendix	single	5
4	Lv Xing et al. 2022	M/60	Chronic Colitis	1 cm	Base	single	1
5	Rocanti et al. 2013	F/49	Irritable Bowel Syndrome	0.2 cm	Tip of appendix	single	6
6	Zoccali et al. 2011	M/19	Acute Appendicitis	3.5 cm	Mid appendix	single	7

**Table 2:** Baseline biochemistry.

Parameter	Result
Hemoglobin	13.2 g/dl
Mean Corpuscular Volume (MCV)	87 fl
White Cell Count	6.8 ×10^9^/l
C-Reactive Protein (CRP)	2.1 mg/l
Serum Creatinine	0.8 mg/dl
Blood Urea Nitrogen (BUN)	13 mg/dl
Alanine Transaminase (ALT)	22 U/l
Aspartate Transaminase (AST)	19 U/l
Alkaline Phosphatase (ALP)	75 U/l
Total Bilirubin	0.6 mg/dl

## Discussion

Granular cell tumors (GCTs) are often found incidentally during screening colonoscopies. They manifest as yellowish-white nodules or polyps with normal overlying mucosa. These tumors most commonly occur in the skin [3], oral cavity, digestive tract, and subcutaneous tissue, but have also been documented in other anatomical locations, including the breast, bladder, thyroid, nervous system, and respiratory system [4]. Within the gastrointestinal tract specifically, GCTs can occur in several regions, such as the esophagus, colon, stomach, and appendix [5]. Granular cell tumors account for approximately 0.5% of all soft tissue tumors, highlighting their rarity in clinical practice. Although most GCTs are benign and present as solitary lesions, about 2% exhibit malignant characteristics. Between 5% and 10% of GCTs are present as multiple rather than solitary tumors. While GCTs can metastasize hematogenously and via lymphatics—most frequently to the lungs, lymph nodes, and bones—the overall incidence of metastasis remains low.

In the GI tract, most GCTs are asymptomatic. When symptoms do occur, tumors in the appendix may mimic conditions such as acute appendicitis, irritable bowel syndrome, or chronic colitis, as shown in our literature review table (Table 1). The tumor size ranges from 0.2 cm to 3.5 cm, with presentations varying from incidental findings to symptoms similar to common GI conditions—highlighting the diagnostic challenge of GCTs. Our case adds to this literature by describing a malignant GCT at the appendiceal orifice in an asymptomatic patient. Endoscopy alone is typically inadequate for detecting submucosal tumors; instead, endoscopic ultrasonography offers more accurate assessment of GI wall layers and tumor origin. Definitive diagnosis is usually obtained via histology, and standard cold-biopsy forceps are generally sufficient for sampling [3]. The Fanburg-Smith criteria were used to distinguish benign from malignant GCTs in our patient. A tumor is considered malignant if it meets at least three of the following: increased pleomorphism, high nuclear-to-cytoplasmic ratio, necrosis, ≥ 2 mitoses per 10 high-power fields, tumor cell spindling, and vesicular nuclei with prominent nucleoli.

Small lesions (< 1 cm) can be monitored conservatively, while larger lesions—especially those > 4 cm—exhibit significant malignant behavior and require surgical resection. Endoscopic removal has been successful for lesions up to 2.6 cm. Techniques such as biopsy forceps or standard snare polypectomy are effective for lesions < 1 cm. Larger lesions warrant advanced procedures: endoscopic mucosal resection, endoscopic submucosal dissection, or surgical resection such as hemicolectomy, as performed in our case [3]. Notably, malignant GCTs have a 40% mortality rate and a high recurrence risk [6]. Consequently, early detection is critical. As in our patient—who was asymptomatic—early histopathology allowed for timely right hemicolectomy before complications arose. Hence, this report emphasizes the importance of histopathology and a high index of suspicion for tumors like GCTs in unusual locations, such as the appendix.

In our patient, MALT hyperplasia and focal ileitis with ulceration were incidental, benign findings unrelated to the GCT. MALT hyperplasia reflects chronic antigenic stimulation, while focal ileitis may result from transient inflammation or mild infection. Given the absence of GI symptoms, autoimmune disease, or chronic infection, no additional intervention was needed beyond monitoring. This underscores the importance of correlating incidental histopathology with clinical context. Granular cell tumors are rare, comprising a small percentage within the GI tract—and even fewer in the appendix. Our case underscores the pivotal role of early detection in asymptomatic patients. Routine colonoscopy and histopathology uncovered a malignant GCT at the appendiceal orifice that might have gone unnoticed. Aggressive surgical intervention ensured complete resection and the prevention of potentially lethal outcomes, reinforcing the need for vigilance in examining submucosal nodules—especially in patients with a family history of malignancy. Finally, this case highlights that histopathology is essential for identifying malignancy, and that timely surgical management can significantly improve prognosis. Early intervention remains the cornerstone of minimizing adverse outcomes in malignant GCTs.

## Consent

Written informed consent was obtained from the patient for publication of this case report and any accompanying details or images.

## Guarantor

Hassan Aryani accepts full responsibility as the guarantor of this manuscript, ensuring the integrity of the data and the accuracy of the presented analysis and conclusions.
